# Standardized and validated training to support the charge nurse: Research protocol

**DOI:** 10.1002/nop2.1727

**Published:** 2023-03-24

**Authors:** Maripier Jubinville, Caroline Longpré, Éric Tchouaket Nguemeleu

**Affiliations:** ^1^ Department of Nursing Sciences Université du Québec en Outaouais Saint‐Jérôme QC Canada

**Keywords:** charge nurse, clinical governance, research protocol, skills, training, transfer of training

## Abstract

**Aim:**

To develop a standardized training for charge nurses.

**Design:**

A developmental research design divided into three parts will be undertaken.

**Methods:**

(1) A scoping review will be used to develop standardized training focusing on charge nurse skills and sub‐skills; (2) a Delphi review with nurses, managers and researchers will validate the content of the training; content validity will be assessed over sufficient rounds of review to obtain a content validity index of over 0.7 and (3) a cross‐sectional study will pilot test the training with 30 charge nurses.

**Results:**

This study will describe the development of updated and empirically validated training to be systematically implemented in healthcare institutions and offered to charge nurses when they begin.

## 1 INTRODUCTION

A charge nurse (CN) performs a clinical‐administrative management position that requires specialized skills (Plourde, 2012). To obtain and promote these skills, CNs require specific training, yet little exists in practice. This lack of training has implications on three levels for (1) the healthcare institution; (2) the CN and (3) the healthcare users. Although some training exists, it tends to be incomplete, not empirically validated, lacking appropriate mechanisms for the transfer of training, and not systematically offered in healthcare institutions. These elements show the importance of developing training such that CNs can receive the support to perform optimally. The main objective of this study is to develop standardized, comprehensive, validated training that addresses the limitations of existing training, integrates the transfer of training from theory to clinical practice, and can be formally included in the organizational structure of healthcare institutions.

## 2 BACKGROUND

A CN is defined as a nurse who performs a clinical‐administrative management position in a healthcare institution to support their unit manager. In Quebec (Canada), there are approximately 6630 CNs, which represent 8.47% of the total number of active nurses with the full right to practice (Ordre des infirmières et infirmiers du Québec, 2021). Plourde (2012) reported that CNs appear to have sub‐optimal levels of five skills that are nevertheless required for the position: leadership; interpersonal communication; clinical and administrative caring; problem‐solving and knowledge and understanding of the healthcare environment. These are broken down into sub‐skills and allow the CN to assume the diverse roles and responsibilities that define the position. To the best of our knowledge, since 2012, no research has addressed the specific integration of the CN skills and sub‐skills outlined by Plourde (2012). However, these have been used in Turmel Courchesne's (2015) qualitative study, where hospital healthcare staff reported that, in general, these skills correspond well to the reality of the CN. Different strategies to support new CNs exist and are offered; an orientation program as described by Sheets (2015) is one example. Despite this, these support strategies did not strengthen CN skills (Lessard, 2018). Training represents one of the most common methods to acquire, maintain and renew CN's knowledge and skill set (Rivard & Lauzier, 2013). Providing training early on supports the development of essential skills, contributes to healthcare user satisfaction and the quality and safety of care (Connelly et al., 2003; Normand et al., 2014; Plourde, 2012; Spiva et al., 2020).

The specific skill set of the CN is not commonly taught in nursing programs, nor when a CN takes on their position. This lack of training has implications on three levels: (1) for the healthcare institution, as the CN may manage their workflow poorly and thus be unable to provide optimal supervision or clinical support to staff, physicians and patients (Armstrong & Hedges, 2006; Connelly et al., 2003); (2) for the CN, as their role will be unclear, which will lead to a lack of understanding of the essential skills needed and how to acquire them to perform well, which will negatively affect their leadership capabilities (Armstrong & Hedges, 2006; Lawrence & Richardson, 2014) and (3) on the users, as this will negatively influence the continuity and quality of care (Armstrong & Hedges, 2006; Connelly et al., 2003). Without appropriate training, a gap persists between the complex requirements of this position and the ability to perform it in practice (Spiva et al., 2020).

Therefore, to enable optimal CN support, training that facilitates a transfer of training is required. Transfer of training can be defined as the extent to which trainees apply the skills, knowledge and attitudes learned in training to their work (Baldwin & Ford, 1988). Optimizing this process involves considering three training inputs: the characteristics of the trainees, the training design and the work environment (Baldwin & Ford, 1988). Positive transfer occurs when work performance is enhanced through the application of these skills and new knowledge, generalized to the new work context and maintained over time (Rivard & Lauzier, 2013). Rivard and Lauzier (2013) specify that when designing training for professionals such as the CN, it is important to consider a model that will promote the transfer of training from theory to clinical practice. These same authors state that it is essential to evaluate the training to assess whether the results: are in line with previously identified objectives; measure the impact of training; identify aspects that need to be improved and provide feedback to the trainees. Finally, the necessary resources and time required for training must be considered in the organizational structure of healthcare institutions in order to promote systematic delivery (Rivard & Lauzier, 2013). All the above‐mentioned components (required skills, a transfer of training model, and the organizational structure of healthcare institutions) are described by the clinical governance model of Brault et al. (2008).

In a narrative review undertaken by our team, 29 articles on CN training were retrieved from the scientific literature (Jubinville et al. n. d). However, these studies have limitations in their clinical governance model (Brault et al., 2008).

### 2.1 Required skills: Training content

In general, training has not been developed based on empirical research. For example, training design and delivery were not described by Sheets (2015), which may lead the reader to question its validity. Some training does not include all the skills required by the CN position as named by Plourde (2012). For example, Bateman (2020) only address leadership skills. If the required skills and knowledge are not provided, a CN may not know how to gain them, and job performance will suffer. Most articles did not specify if training material was used, nor was it found in the publication annexes. This makes it difficult to critically analyse the content and determine whether the training is appropriate.

### 2.2 The process of transfer of training from theory to clinical practice

From this narrative review, the literature does not adequately describe training that would successfully promote the transfer of training into clinical practice. There is no mention of whether the necessary inputs (trainee characteristics, training design and work environment) are considered in the development and delivery of training. Although, for example, Spiva et al. (2020) describe pedagogical strategies used, other inputs are not described. This may result in less‐than‐optimal integration and transfer of the training for CNs.

### 2.3 Delivering training in the organizational structure of healthcare institutions

Currently, it is not standard practice for healthcare institutions to offer training to newly appointed CNs in preparation for their position. One exception is a study describing training developed and systematically delivered in an ambulatory setting, which resulted in increased CN self‐confidence and knowledge (Andronico et al., 2019). Delivering training requires many resources: human resources, including liberating trainees from their duties, finding their replacement and suitable trainers; material resources such as audiovisual equipment and course materials and financial resources to cover salaries and the materials required (Rivard & Lauzier, 2013). These resources, along with the time needed to organize a training session may explain why training is not systematically given. The length of time training requires must be considered. For example, Normand et al. (2014) describe training that lasts 42 h, which may not be realistic due to the resources required. In this sense, it is necessary to develop training that is viable, accessible and easily implemented in the organizational structure of healthcare institutions.

Essential components of CN training are partially or completely lacking from the literature reviewed. The use of a governance model for the development of training is essential to: guide the content that will be covered and the pedagogical material that will be deployed; integrate structural mechanisms that promote the transfer of training from theory to clinical practice in order to support the integration of new knowledge and use a formal healthcare institutional structure to ensure that training is systematically provided when CNs are hired.

## 3 METHODS

### 3.1 Aims

The main objective of this study is to develop a standardized and validated training that addresses the limitations of existing training, integrates the transfer of training from theory to clinical practice and can be included in the organizational structure of healthcare institutions. The specific objectives of this study are to: (1) develop a standardized training to address the skills and sub‐skills expected of the CN; (2) using a Delphi review consult a group of experts to validate the content of the training, the relevance of the suggested transfer of training and the methods proposed (e.g. resources required, length of the training) to include in the organizational structure of healthcare institutions for implementation and (3) run a pilot test to validate these same elements and verify whether this training can positively influence the skills and knowledge of the CN.

### 3.2 Research questions

Three research questions will be explored:

1. To what extent can the skills and sub‐skills of a CN be improved through the provision of standardized training?
2. Will CNs apply new knowledge in clinical practice if they receive transfer of training from theory to practice?
3. Are the proposed methods of including training in the organizational structure of healthcare institutions conducive to improving its accessibility?

### 3.3 Conceptual framework

**FIGURE 1:**
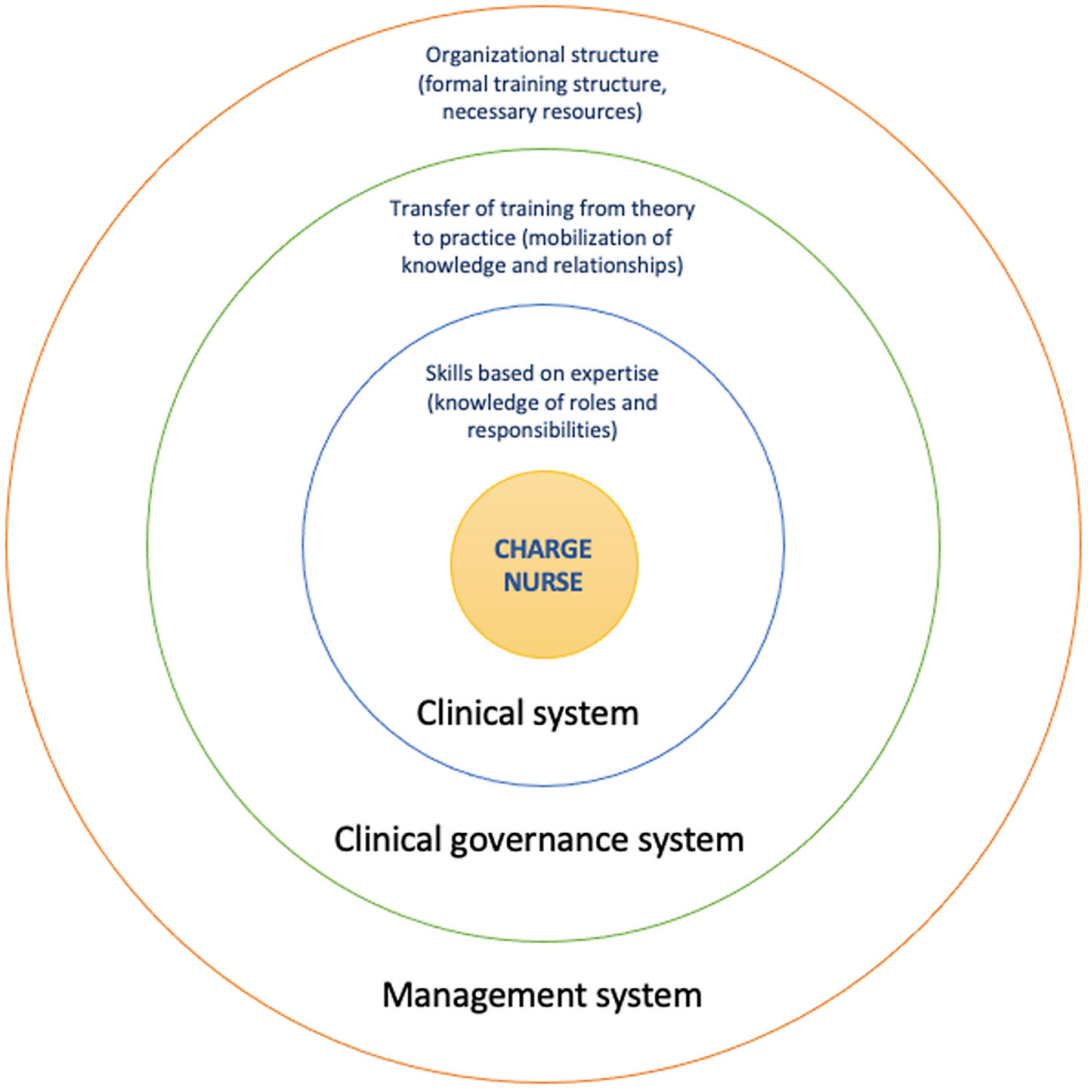
Training model for the charge nurse (TMCN) (adapted from Brault et al., 2008)

This study uses the clinical governance model of Brault et al. (2008), which bridges institutional and professional perspectives. Brault's model simultaneously integrates all the necessary systems to be taken into consideration to strengthen the skills of the CN and has been adapted for a training context for this study (see Figure 1).

Our ‘Training Model for Charge Nurses’ (TMCN) puts forward three components necessary for the development and deployment of CN training: (1) the specific skills of the position; (2) the three training inputs to consider in order to promote the transfer of training from theory to clinical practice and (3) organizational structure of healthcare institutions required to support the systematic delivery to new CNs. Each of these three components has a corresponding part of the interconnected Brault model: (1) the clinical system; (2) the clinical governance system and (3) the management system.

#### 3.3.1 Skills based on expertise (knowledge of roles and responsibilities)

The first component is a part of the clinical system that is the centre of the CN's profession. In this study, the component is operationalized by the five CN skills (Plourde, 2012) which can be broken down into 23 sub‐skills (see Table 1), and are made use of in practice through specific roles and responsibilities.

**TABLE 1:** Charge nurse skills and sub‐skills (Plourde, 2012).

Skills	Leadership	Interpersonal communication	Clinical and administrative caring	Problem‐ solving	Knowledge and understanding of the healthcare environment
Sub‐skills	Knows how to mobilize people	Knows how to listen	Takes a humanistic approach	Has a global vision	Focuses on patients and results
	Facilitates change	Knows how to express themself	Maintains a spirit of openness and altruism	Has the ability to analyse and synthesize	
	Is proactive	Verifies that information is understood by others	Encourages the expression of feelings with empathy	Displays critical judgement and sound decision making	
	Establishes a relationship of trust		Provides support	Has the capacity to create and innovate	
	Knows how to negotiate		Accompanies others in their development	Able to manage conflict	
	Knows how to collaborate			Has coping skills (self‐control, self‐confidence, managing emotions, perseverance).	
	Makes use of the strengths of individuals			Has coordination skills (organized, able to delegate, respects others' fields of practice, exhibits good time management)	

#### 3.3.2 Transfer of training from theory to clinical practice (mobilization of knowledge and relationships)

**FIGURE 2:**
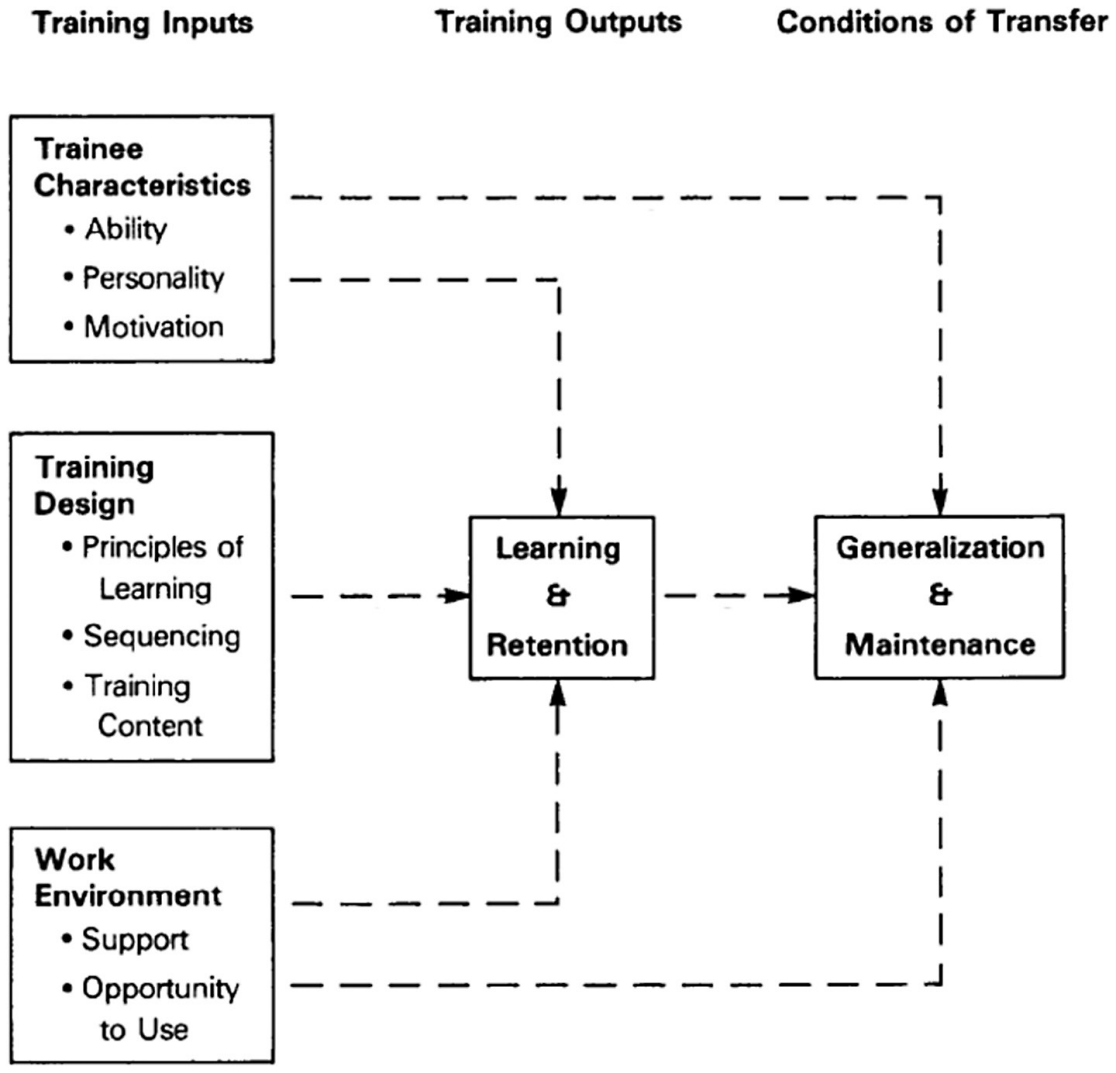
Adapted from the model of transfer process (Baldwin & Ford, 1988).

**FIGURE 3:**
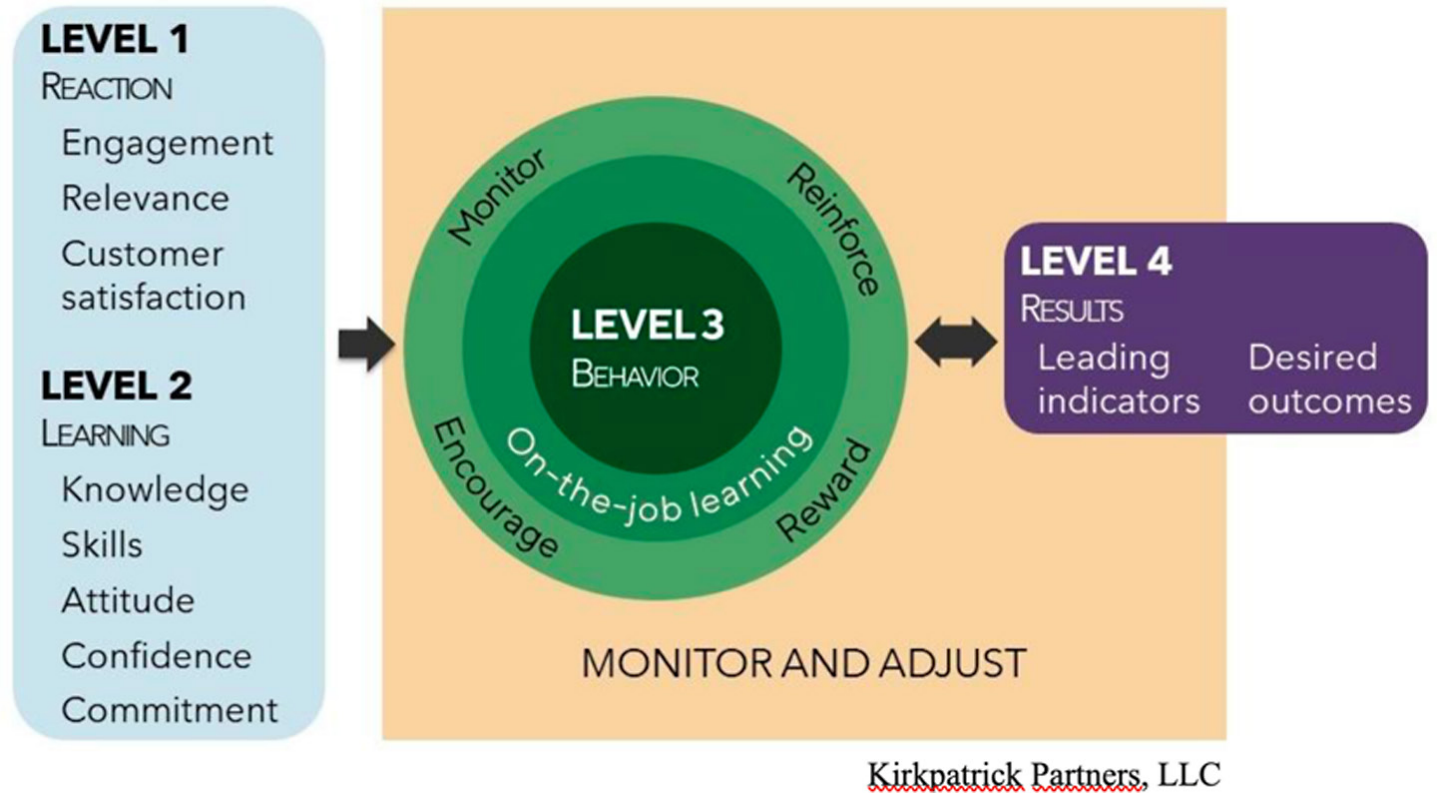
The new world Kirkpatrick model (Kirkpatrick & Kirkpatrick, 2016).

This second component is a part of the clinical governance system that facilitates clinical and management initiatives that are dedicated to the quality of care, clinical excellence and institutional performance (Brault et al., 2008). In this study, it reflects the generalization of knowledge, acquired in training, to the workplace, as well as the integration of new relationships with colleagues or managers that permit the application of the knowledge learned. Transfer of training is operationalized by Baldwin and Ford (1988; Figure 2) and Kirkpatrick and Kirkpatrick (2016; Figure 3). Baldwin and Ford (1988) describe the inputs necessary (trainee characteristics, training design and work environment) to learn and retain information, as well as what generates and helps maintain new knowledge. Training is said to be successful when knowledge is applied at work and maintained over time. Kirkpatrick and Kirkpatrick (2016) present four levels to be considered when evaluating training. These include the following: (1) reactions; (2) learning; (3) transfer of training from theory to clinical practice (behaviour) and (4) results. Integrating this model will help highlight the side benefits of training and identify adjustments required for successful transfer. These complementary models will make it possible to evaluate the training and promote its transfer.

#### 3.3.3 Organizational structure (formal training structure, necessary resources)

This last component is the part of the management system that supports professionals through the provision of formal supervisory structures (Brault et al., 2008). This means that healthcare institutions must put in place a structure that will allow for the systematic delivery of training when a new CN arrives. Indeed, CNs that received standardized training at hire emphasized how it clarified their role and made them feel confident in their position (Lessard, 2018).

In sum, the TMCN model provides a relevant model for this study. This integrative framework serves as a foundation for the design and delivery of training for the CN to meet clinical governance methods necessary to strengthen their skills.

### 3.4 Study design

A developmental research design will be used. Developmental research is defined as the analysis of the object development process (training) including the design and the pilot test of the object, taking into account the data collected at each phase of the process and the existing scientific literature (Loiselle & Harvey, 2007). The pilot test is used to analyse the interactions between the intervention under development (training) and the people for whom the intervention is designed (here, the CN) in order to improve the prototype and to inform its development and possible future modifications (Loiselle & Harvey, 2007). This study will be subdivided into three parts to address each of our research objectives. First, a scoping review will be used to research and inform the development of the CN training by June 2023. Second, a Delphi review will be used to validate the content of the training and the proposed transfer of training by October 2023. Finally, we will pilot test the developed training with a cohort of 30 CNs, using a cross‐sectional design, to assess feasibility and the impact on skill set strengthening by December 2023.

#### 3.4.1 Part 1: Scoping review for the development of a training

This scoping review will follow the framework proposed by the Joanna Briggs Institute (JBI; Peters et al., 2020) which encompasses nine steps:

Step 1: Defining and aligning the objective and questions

The objective of the scoping review is to review the literature that addresses any of the five CN skills reported by Plourde (2012). The articles analysed will help describe and define skills, set out how they are operationalized in practice, and what their impact on the institution of work and the quality of care may be. To be retained for this analysis, articles must have a positive response to any of our research questions:

1. Do the articles describe the skills required for the position of CN?
2. Do the articles highlight key training elements for developing CN skills?
3. Do the articles indicate any positive or negative impacts (e.g. on filling the CN position, on the institution where the CN works) that are associated with these skills?

Step 2: Developing and aligning the inclusion criteria with the objective and questions

The inclusion criteria for the scoping review are in line with the objective and research questions of Step 1. Articles must be written in French or English and have been published since 2000. The selected literature will meet the scoping review criteria defined by the PICOT, which is consistent with the TMCN clinical system, and includes: the study population (P); the intervention or treatment discussed in the review (I); the comparison if any, (C); the outcomes (O) and the time period during which the data will be collected (T).

Step 3: Describing the planned approach to evidence searching, selection, data extraction and presentation of the evidence

A literature search will be conducted using the CINAHL, MEDLINE, Science Direct and Cairn databases. The following keywords (MeSH and associated descriptors) will be combined to create a search strategy using AND and OR Boolean operators: *assistante infirmière‐chef, assistante du supérieur immédiat*, charge nurse, first‐line nurse manager, matron, ward leader, ward sister, leadership, communication, caring, problem‐solving and work environment. Also, the grey literature (non‐scientific, theoretical, conceptual, dissertations, theses and conference presentations) will be consulted in ProQuest dissertations and thesis global, and in Google Scholar.

Step 4: Searching for the evidence

First, a search in the CINAHL and MEDLINE databases will be completed using the original search strategy. A research librarian and two scoping review experts will then be consulted and based on the articles retrieved, a keyword analysis will be performed to create a revised search strategy. All four databases will then be queried using this updated strategy.

Step 5: Selecting the evidence

After eliminating duplicates and verifying the language, articles will be exported to Rayyan web platform (Ouzzani et al., 2016). Two team members will ensure content validity by reading the titles and abstracts of 10% of all retrieved articles to ensure that the keywords used obtained the expected results and that searches were performed in a similar manner. Following this, two team members will read the titles and abstracts of all articles to determine their relevance. Relevant articles will then be read in their entirety and assessed by both team members independently for inclusion or exclusion. In the case of a conflict, a third team member will read the article in its entirety and determine whether it should be included or excluded.

Step 6: Extracting the evidence

The selected articles will be independently analysed by two team members using standardized data extraction tables. Data will then be entered into Excel spreadsheets. The results will be categorized according to our first research question that focuses on the skills required of CNs (Plourde, 2012).

Step 7: Analysis of the evidence

Data analysis will be conducted using Saldaña (2016) and Miles et al. (2019) methods that consist to: (1) design and map the skills; (2) define the skills, how they are operationalized in practice, and their impact and (3) synthesize the results in order to have all the important elements for training development.

Step 8: Presentation of the results

The results will be summarized and presented in several forms, including tables, diagrams and evidence maps. The training will be divided into sections corresponding to the CN's position generally (e.g. description of the CN, how the position evolved), their skills and sub‐skills.

Step 9: Summarizing the evidence in relation to the purpose of the review, making conclusions and noting any implications of the findings

A summary of the results will be made in relation to this study's objective, conclusions will be drawn, and potential implications of these results will be shared. The results of this study will be published in a peer‐reviewed journal and presented in scientific conferences. In addition, findings will be presented to provincial Directors of Nursing in Quebec (Canada), describing how skills training for the CN will be developed.

#### 3.4.2 Part 2: A Delphi review by experts to ensure training validity

The training developed will be validated through a Delphi review which required consensus of experts (Ekionea et al., 2011). This method consists of seven steps:

Step 1: The number of experts is determined; for this study, 18 experts will be consulted (Turoff, 2002). These experts will be equally divided among (a) a clinical setting, consulting CNs (*n* = 6) and managers (manager, assistant director, senior consultant; *n* = 6) who have held a position for at least 6 months on a hospital unit; (b) researchers from a university setting in Québec (*n* = 6), holding a position as a professor or lecturer and having expertise related to the format or content of the training. Non‐probability sampling will be used to ensure a diversity of respondents from each setting and for each expertise.
Step 2: An email describing relevant experts to be recruited with pertinent details of the study will be prepared for (1) the clinical setting, where it will be sent to nursing directors; and (2) the university setting, where it will be sent to researchers (professors and lecturers).
Step 3: Experts will be individually contacted and presented with information about the study, after which they will be asked if they would like to participate.
Step 4: Experts who express interest will be invited to participate in the study. They will be sent information and consent forms and asked to provide written informed electronic consent.
Step 5: To evaluate the training, the experts will receive a questionnaire developed by the research team and administered through LimeSurvey. Participants will evaluate: the training content; the relevance of the transfer of training and the organizational structure methods proposed to administer the training. Experts will be able to evaluate the content on a Likert scale with four response options from 1 = ‘not relevant’ to 4 = ‘very relevant without corrections’ as shorter Likert scales have produced stable findings in a previous Delphi study (Tchouaket Nguemeleu et al., 2020). Also, space will be provided for open‐ended commentary.
Step 6: The feedback will be consolidated and analysed.

A content validity index (CVI) analysis will be performed. A CVI up to 0.7 allows for acceptance of the content without modification, while a CVI of less than 0.7 requires modification. Response feedback is given to the team and a new round will ensue, thus the number of rounds will be determined by the responses until a CVI equal to or greater than 0.7 is achieved (up to a maximum of three rounds; Turoff, 2002).

Step 7: In a final step, the content will be adjusted and finalized based on expert feedback.

#### 3.4.3 Part 3: Descriptive cross‐sectional study to pilot test the CN training

A cross‐sectional study will be undertaken in four steps:

Step 1: The subjects and study setting will be selected. As per the TMCN, the target population for this study are CNs (*n* = 30) working at a hospital healthcare unit in Québec (Canada). The CNs will be recruited from similar work environments such that the examples presented during training are appropriately adapted and the content will be more representative of their shared professional experiences. CNs will be recruited through non‐probability convenience sampling. To be eligible, participants will have to hold a valid Quebec Order of Nurses (OIIQ) licence, work in a hospital and occupy a CN position in any capacity. We will exclude CNs currently under competency, ethical or disciplinary review. A list of CNs will be requested from the study setting's human resources department and a recruitment poster describing the study details will be sent internally to CNs. For those who express interest, information, consent and confidentiality forms will be sent via the LimeSurvey platform. If a response to the invitation has not been received within 2 weeks, one email reminder will be sent.
Step 2: The training will then be pilot tested. The training will last 7 h and will be offered at three different times to ensure sessions are accessible for all participants. Due to COVID‐19, government recommendations suggest training should be offered with appropriate health measures in place. Participants will be offered virtual or in person training and will respect the required health measures. In order to optimize transfer of training, a maximum sample size of 12 participants is recommended (Rivard & Lauzier, 2013). The same researcher will provide all training sessions to ensure consistency and reliability across sessions.
Step 3: To evaluate the training, the CNs will complete a self‐administered LimeSurvey questionnaire developed by the research team and validated by 5 CNs. Questions will offer a Likert scale with four response options from 1 = ‘totally disagree’ to 4 = ‘totally agree’. The questionnaire will ask for socio‐demographic information (e.g. ‘How many years of experience do you have as a CN’?) as well as questions to assess each of the components of the TMCN. Examples of questions on training content include: ‘The content presented in this training is applicable to my CN position’; for the relevance of the transfer of training ‘The exercises and activities carried out will allow me to more easily transfer what I learn in training to my practice’, and for the relevance of the organizational and structural methods proposed to implement the training ‘The length was adequate’. The questionnaire will ask whether the training can strengthen CN skills, for example regarding leadership: ‘Following my participation in this training, my leadership skills have been strengthened, for example, I will be able to carry out my roles and responsibilities more effectively, and better assume my overall position’. Also, the feasibility and acceptability will be assessed with questions such as: ‘It was difficult to get my manager to release me to participate in this training’. A space to provide additional feedback will be available for each question.
Step 4: Data analysis: SPSS software version 26 will be used to analyse the data. Descriptive and bivariate analyses will be done using parametric and non‐parametric tests as appropriate. These analyses will describe the needs and preferences of CNs. Content analyses will be conducted on the comments made by the participants.

### 3.5 Ethical considerations

Ethical approval for this research was obtained from the Research Ethics Board of the university where the research is taking place (June 2021, # 2021–1261) and the Research Ethics Board of the hospital in this study (December 2021, # 2021‐333_168_MP). Ethical principles in research will be respected. All participants will be made aware of the rationale of the study, time involved, inclusion criteria and the ability to withdraw from the study at any time without consequences. At each stage of the study, all information will be transmitted to the participants so that they can ask questions. They will also be informed of the advantages of participating in the pilot test (such as access to expert‐validated training) and the disadvantages (the time required to obtain training). Participants in this study will provide written electronic free and informed consent and will sign a confidentiality form prior to their participation. Managerial approval for the release of CN participants will be sought prior to conducting the training. The confidentiality of the data collected will be ensured by assigning a numeric code to each participant. Finally, the data collected will be saved on the university's server and stored on a password‐protected computer at the university. All data will be confidentially and permanently deleted 5 years after the end of the study.

### 3.6 Validity and reliability/rigour

To ensure a rigorous research process four core elements will be put in place: (1) using a comprehensive framework to fill gaps in the scientific literature on increasing the skills of an incoming CN; (2) performing a scoping review to ensure evidence‐based data is used for training development; (3) using a Delphi review to reinforce the internal validity and scientific rigour of the training's content and (4) pilot testing the CN training in order to ensure the quality of the training with validated and evidence‐based content. The use of a rigorous process to meet the specific objectives of this research increases internal, external, construct, content and instrumental validity.

## 4 DISCUSSION

This research project will lead to the creation of standardized training specifically geared to the CN. We therefore assume that more support in terms of training for CNs will enable them to optimize their practice by better mastering their skills, as well as the general functioning of their unit and this will ultimately have an impact on staff and user satisfaction, on the quality and safety of the care and on the work environment. This study will enable CNs to increase the efficiency of their clinical‐administrative management skills within their practice. By developing strong leadership skills and working alongside their manager, CNs will be better able to achieve the institutional objectives targeted by the governance structure and strategic planning that is in place in their unit. In the context of nursing research, this study will contribute to the literature on optimization of the CN position. In addition, this project will generate evidence‐based data that will provide a solid scientific foundation for the development of future research and will strengthen expertise in this field. Thus, the field of nursing will benefit from the developed training as it will clearly define the position and inform the training and practice of CNs. In summary, this research will help fill the gap in the scientific literature for training that is comprehensive, evidence‐based, standardized, validated by experts and tested with CNs. Steps taken to test its validity will ensure that this training can be generalized to all CN working in a healthcare institution (with examples adapted according to the healthcare sector in which the CN are working) and be integrated into their clinical governance to be offered systematically to all new CNs in Québec, Canada and internationally.

This research has limitations. Training will be developed for CNs working in hospital units in Québec, Canada. It is possible that certain details of the training will be specific to the job description of CNs working in this setting and may require adjustments to the material for other settings. For CNs working outside of Québec the content will require context‐specific editing to ensure it accurately represents the roles and responsibilities of CNs in other regions. Also, healthcare institutions differ in terms of financial and human resources, culture, work systems and user characteristics; so these variables will affect the study outcome and thus render the findings not completely generalizable to other hospitals. Finally, the pilot test will require replication on a larger scale and in different work contexts to ensure its validity and generalizability.

## AUTHOR CONTRIBUTIONS

M.J., C.L., E.T.N.: Made substantial contributions to conception and design, or acquisition of data, or analysis and interpretation of data; M.J., C.L., E.T.N., Involved in drafting the manuscript or revising it critically for important intellectual content; M.J., C.L., E.T.N.: Given final approval of the version to be published. Each author should have participated sufficiently in the work to take public responsibility for appropriate portions of the content; M.J., C.L., E.T.N.: Agreed to be accountable for all aspects of the work in ensuring that questions related to the accuracy or integrity of any part of the work are appropriately investigated and resolved.

## CONFLICT OF INTEREST STATEMENT

The authors declare no conflict of interest.

## PATIENT AND PUBLIC INVOLVEMENT

This research was undertaken without patient or public involvement. They were not invited to contribute to the writing nor to the editing of this study protocol for readability or accuracy.

## Data Availability

The data that support the findings of this study are available from the corresponding author upon reasonable request.
